# Climate suitability analyses compare the distributions of invasive knotweeds in Europe and North America with the source localities of their introduced biological control agents

**DOI:** 10.1002/ece3.10494

**Published:** 2023-09-12

**Authors:** Jeremy C. Andersen, Joseph S. Elkinton

**Affiliations:** ^1^ Department of Environmental Conservation University of Massachusetts Amherst Amherst Massachusetts USA

**Keywords:** classical biological control, environmental niche modeling, *Fallopia*, genotype × environment interactions, *Reynoutria*

## Abstract

Climate suitability analyses based on ecological niche modeling provide a powerful tool for biological control practitioners to assess the likelihood of establishment of different candidate agents prior to their introduction in the field. These same analyses could also be performed to understand why some agents establish more easily than others. The release of three strains of *Aphalara itadori* (Shinji) (Hemiptera: Pysllidae), each from a different source locality in Japan, for the biological control of invasive knotweed species, *Reynoutria* spp. Houtt. (Caryophyllales: Polygonaceae), provides an important opportunity to compare the utility of climate suitability analyses for identifying potential climate‐based limitations for successful biological control introductions. Here, we predict climate suitability envelopes for three target species of knotweed in Europe and two target species of knotweed in North America and compare these suitability estimates for each of these species to the source localities of each *A. itadori* strain. We find that source locality of one strain, the Kyushu strain, has little‐to‐no suitability compared to other locations in Japan based on knotweed records from Europe, supporting an earlier study based on North American Japanese knotweed records. The source locality of a second strain, the Murakami strain, was predicted to have medium‐to‐high suitability based on records of knotweeds from North America. In contrast, European records of *Reynoutria* × *bohemica* Chrtek & Chrtková and *Reynoutria sachalinensis* (F. Schmidt) Nakai predicted no suitability for this locality compared to other locations in Japan, while European records for *Reynoutria japonica* Houtt. predicted low suitability. The source locality of the final strain, the Hokkaido strain, was predicted as having medium‐to‐high suitability based on knotweed records of all examined species from both North America and Europe.

## Graphical abstract

Climate suitability analyses for invasive knotweed species based on European and North American locality information predict no‐to‐low suitability for two of the three source localities of the natural enemy *Aphalara itadori*. We believe this climate mismatch may be inhibiting the establishment of this important biological control agent.

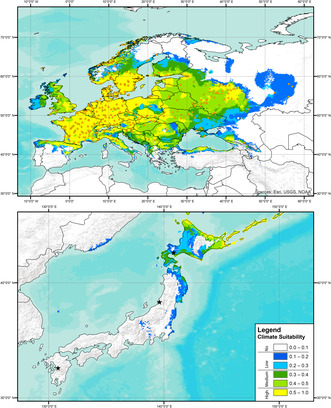

## 1 INTRODUCTION

Biological control is a safe and cost‐effective approach for the landscape‐wide management of weedy species (Van Driesche et al., 2010). On numerous occasions, insects and pathogens have been identified and promoted to reduce the environmental and economic damages of weeds in locations from across the globe (Van Driesche, 2012). Despite concerns about the non‐target effects of biological control agents (Barratt et al., 2010; Howarth, 2000; Simberloff, 2012), modern biological control programs implement a system of safe guards to reduce unwarranted damage to non‐target species—particularly when the agent is being introduced from a novel region through a practice known as classical biological control (Heinz et al., 2016; Messing, 2001). As a result, the use of biological control as an alternative to other labor and chemically intensive methods is increasingly becoming a part of both conservation and organic management practices (Baker et al., 2020; Van Driesche et al., 2016).

The control of invasive knotweed species, *Reynoutria* spp. Houtt. (Caryophyllales: Polygonaceae), has received much attention, with cultural, mechanical, and chemical control options all being implemented (e.g., Delbart et al., 2012; Kadlecová et al., 2022; Martin et al., 2020). Interest has also been directed toward harvesting knotweeds, as the plants have unique chemical properties (Metličar et al., 2021; Metličar & Albreht, 2022) and may themselves be an important source for biopesticides (Dara et al., 2020). However, for landscape‐wide efforts biological control is likely the most effective strategy and as such an international effort was established to identify and promote the natural enemy, *Aphalara itadori* (Shinji) (Hemiptera: Aphalaridae), which was observed feeding and causing damage upon wild populations of *Reynoutria japonica* Houtt. on the Japanese island of Kyushu in 2004 (Shaw et al., 2009). Prior to field releases, a laboratory‐reared population of the Kyushu strain of *A. itadori* was then used for host‐range testing and candidate biological control reviews were conducted (Grevstad et al., 2013; Shaw et al., 2009, 2011), resulting in the first approved biological control agent in the European Union (Shaw et al., 2009). A second population of *A. itadori*, feeding on *Reynoutria sachalinensis* (F. Schmidt) Nakai, was subsequently collected in 2007 near Lake Toya on the Japanese island of Hokkaido, and similarly brought to the laboratory for host‐range testing and candidate biological control review (APHIS, 2020). Both strains were subsequently approved for release in Europe and North America, and recently a third strain, the Murakami strain was identified from near the Japanese city of Murakami and has been released in the Netherlands against *Reynoutria* × *bohemica* Chrtek & Chrtková (Camargo et al., 2022). Review of the Murakami strain for release in North America is currently underway.

As part of the review prior to introduction in North America, climate suitability models for the Kyushu strain and the Hokkaido strains were developed using the software program CLIMEX (Hearne Software, Melbourne, Australia). These models predicted a strong climate match for both the Kyushu and Hokkaido strains to potential release locations across North America (Grevstad et al., 2012). However, despite this predicted climate match, there have been no documented accounts of establishment of this species anywhere it has been released. Note: here we use establishment to indicate a self‐sustaining population that is present in a location for at least three consecutive years without importation or release of additional individuals. We choose to use this more conservative definition, though “establishment” has historically been reported in the literature after only 1 year (see Van Driesche et al., 2008). Unfortunately to date in locations where the psyllids have been released and individuals have been observed in the field during post‐release monitoring, neither reduction in plant densities nor biomass have been observed. We previously suspected that environmental constraints might be limiting the success of the Kyushu strain in North America (Andersen & Elkinton, 2022), and noted a poor climate match to the source locality of the Kyushu strain based on North American records of *R. japonica* using a different modeling approach, MaxEnt (Phillips et al., 2006; Phillips & Dudik, 2008). The MaxEnt models based on *R. japonica* records did, however, predicted medium‐to‐high suitability for the source localities of the Hokkaido and Murakami strains (Andersen & Elkinton, 2022). Therefore, we were curious as to whether climate suitability estimates conducted in MaxEnt for each target knotweed species based on records from Europe and North America could help provide insights into factors influencing the success of *A. itadori* releases in each region and for each target species of knotweed.

To address this, we collected public records of all three target knotweed species from the Global Biodiversity Information Facility (GBIF) from Europe and North America. Using the MaxEnt software platform, we estimate climate suitability envelopes based on records from the invasive regions of each species, and we compared the predicted suitability of the source locality of each *A. itadori* strain compared with other localities in Japan for each species of knotweed and geographic region combination.

## 2 MATERIALS AND METHODS

The climate suitability analyses presented here follow the methods of Andersen and Elkinton (2022) using locality records obtained from the invaded distributions of knotweed species to predict regions in the native range of these species where candidate biological control agents might be most successfully established. As per Andersen and Elkinton (2022), we used host records (i.e., knotweed records) as a proxy for their specialist parasites (i.e., *A. itadori*), as the records for hosts are often more readily available in public databases (Andersen & Elkinton, 2022; Johnson et al., 2019; Schneider et al., 2022).

Climate suitability analyses were based on the use of published records for all species of *Reynoutria* (knotweeds) obtained from the GBIF database (accessed on September 28th, 2022: GBIF Occurrence Download https://doi.org/10.15468/dl.pdjdh8). This dataset was then filtered to remove all records that lacked geographic locality information and then subdivided by focal species, resulting in one dataset each for *R. japonica*, *R*. × *bohemica*, and *R. sachalinensis*. The species datasets were then further subdivided into geographical bins, with separate bins for samples from North America (those samples located between 0° N, 180° W and 90° N, 20° W) and from Europe and western Asia (those samples located between 0° N, 20° W, and 90° N, 60° E).

To reduce the effects of sampling biases in our analyses we followed the recommendations of Hijmans and Elith (2021). In the R statistical language environment (R Core Team, 2022), we used the packages “raster” (Hijmans & van Etten, 2012) and “dismo” (Hijmans et al., 2015) to randomly select one observation per 1 min × 1 min grid cell within each dataset. The final datasets were then used to independently estimate climate suitability envelopes in MaxEnt v 3.3.3e (Phillips et al., 2006; Phillips & Dudik, 2008) based upon the 5‐min resolution WorldClim v 2.1 dataset (available at https://www.worldclim.org). Jack‐knife analyses were performed on each dataset to measure the relative importance of each climate variable, and results were mapped in ArcGIS v 10.8 (Esri®, Inc., Redlands, CA).

## 3 RESULTS

A total of 249,769 records of *Reynoutria* specimens with locality information (i.e., latitude and longitude coordinates) were downloaded from the GBIF database. These included 232,744 records for *R. japonica*, 11,608 records for *R. sachalinensis*, and 4377 records for *R*. × *bohemica*. After geographic binning, and grid‐based filtering, the European *R. japonica* dataset included 721 records; the European and North American *R. sachalinensis* datasets included 432 and 114 records, respectively; and the European and North American *R*. × *bohemica* included 342 and 92 records, respectively. Model accuracy for the MaxEnt analyses based on the ‘area under the receiver‐operator curve’ (AUC) approach (Merow et al., 2013), indicated that all models had high AUC scores (European *R. japonica* AUC = 0.948, European *R. sachalinensis* AUC = 0.966, European *R*. × *bohemica* AUC = 0.969, North American *R. sachalinensis* AUC = 0.985, North American *R*. × *bohemica* AUC = 0.986). In total, 10 of the 18 BioClim variables provided ≥10% contribution to the MaxEnt model predictions for at least one of the datasets (Table 1). In Europe, five variables contributed ≥10% to the climate suitability envelope for *R. japonica*, four variables contributed ≥10% to the climate suitability envelope for *R*. × *bohemica*, and three variables contributed ≥10% to the climate suitability envelope for *R. sachalinensis*. Two of the 18 variables, Bio 6 (min temperature of the coldest month) and Bio14 (precipitation of the driest month), were included in models for all three focal species. In North America, two variables contributed ≥10% to the climate suitability envelope for *R*. × *bohemica*, and three variables contributed ≥10% to the climate suitability envelope for *R. sachalinensis*. Two of the 18 BioClim variables, Bio 1 (annual mean temperature) and Bio 19 (precipitation of the coldest quarter) were present in models for both focal species (Table 2).

**TABLE 1:** Relative importance of each BioClim climate variable as predicted by Jack‐Knife analyses in MaxEnt for Japanese (*Reynoutria japonica*), Bohemian (*Reynoutria* × *bohemica*), and Giant (*Reynoutria sachalinensis*) knotweeds. Variables contributing ≥10 to each climate suitability envelope are highlighted.

Variable	Europe			North America		
	Japanese	Bohemian	Giant	Japanese^a^	Bohemian	Giant
Bio1—Annual Mean Temperature	8.6	13.8	5.9	21.2	33.3	29.7
Bio2—Mean Diurnal Range	13.7	9.8	7.6	1.3	0.4	0.4
Bio3—Isothermality	8.3	6.8	6.7	2.2	0.2	0.2
Bio4—Temperature Seasonality	0.1	2	0.2	1	6.7	0.7
Bio5—Max Temperature of Warmest Month	6.4	1.2	11.5	0.7	0	0.1
Bio6—Min Temperature of Coldest Month	15.2	11	13.5	0.7	0.9	1.1
Bio7—Temperature Annual Range	1.3	0.7	2.1	6.2	2	1.6
Bio8—Mean Temperature of Wettest Quarter	10	1.2	2	0.2	0.4	0.5
Bio9—Mean Temperature of Driest Quarter	0.6	1.9	0.3	1.5	0.7	1.2
Bio10—Mean Temperature of Warmest Quarter	0.5	10	7.1	1.8	3	9.1
Bio11—Mean Temperature of the Coldest Quarter	1.3	2.9	4.1	16.8	8.7	6.3
Bio12—Annual Precipitation	0.8	0	0.2	23.8	9.5	12
Bio13—Precipitation of Wettest Month	0.3	0	0.2	0	0	0.2
Bio14—Precipitation of Driest Month	12.9	17	28.9	0.1	0.4	0.2
Bio15—Precipitation Seasonality	3.5	6	9.5	2.6	0.5	0.8
Bio16—Precipitation of Wettest Quarter	0	1	0	1.5	0.4	0.8
Bio17—Precipitation of Driest Quarter	15.9	13.7	0.1	6.1	5.2	0.3
Bio18—Precipitation of the Warmest Quarter	0.3	0.7	0.1	1.8	3.7	2.6
Bio19—Precipitation of the Coldest Quarter	0.1	0.3	0.1	10.7	23.9	32.4

^a^ Results for Japanese knotweed in North America are reprinted from Andersen and Elkinton (2022) with permission.

**TABLE 2:** Climate suitability of candidate strains for each target species as determined by the presence or absence of suitable habitat in the geographical vicinity of the source locality for each candidate strain of *Aphalara itadori* against each target species of knotweed (Japanese = *Reynoutria japonica*, Bohemian = *Reynoutria* × *bohemica*, and Giant = *Reynoutria sachalinensis*).

	Kyushu	Murakami	Hokkaido
Europe			
Japanese knotweed	No Suitability	No/Low Suitability	Low/Medium Suitability
Bohemian knotweed	No Suitability	No Suitability	Low/Medium Suitability
Giant knotweed	No Suitability	No Suitability	Low/Medium Suitability
All North American Records			
Japanese knotweed^a^	No Suitability^a^	High Suitability^a^	Medium/High Suitability^a^
Bohemian knotweed	No/Low Suitability	Medium/High Suitability	Low/Medium Suitability
Giant knotweed	No Suitability	Medium/High Suitability	Medium/High Suitability

^a^ Results for Japanese knotweed in North America are summarized from the analyses presented in Andersen and Elkinton (2022).

**FIGURE 1:**
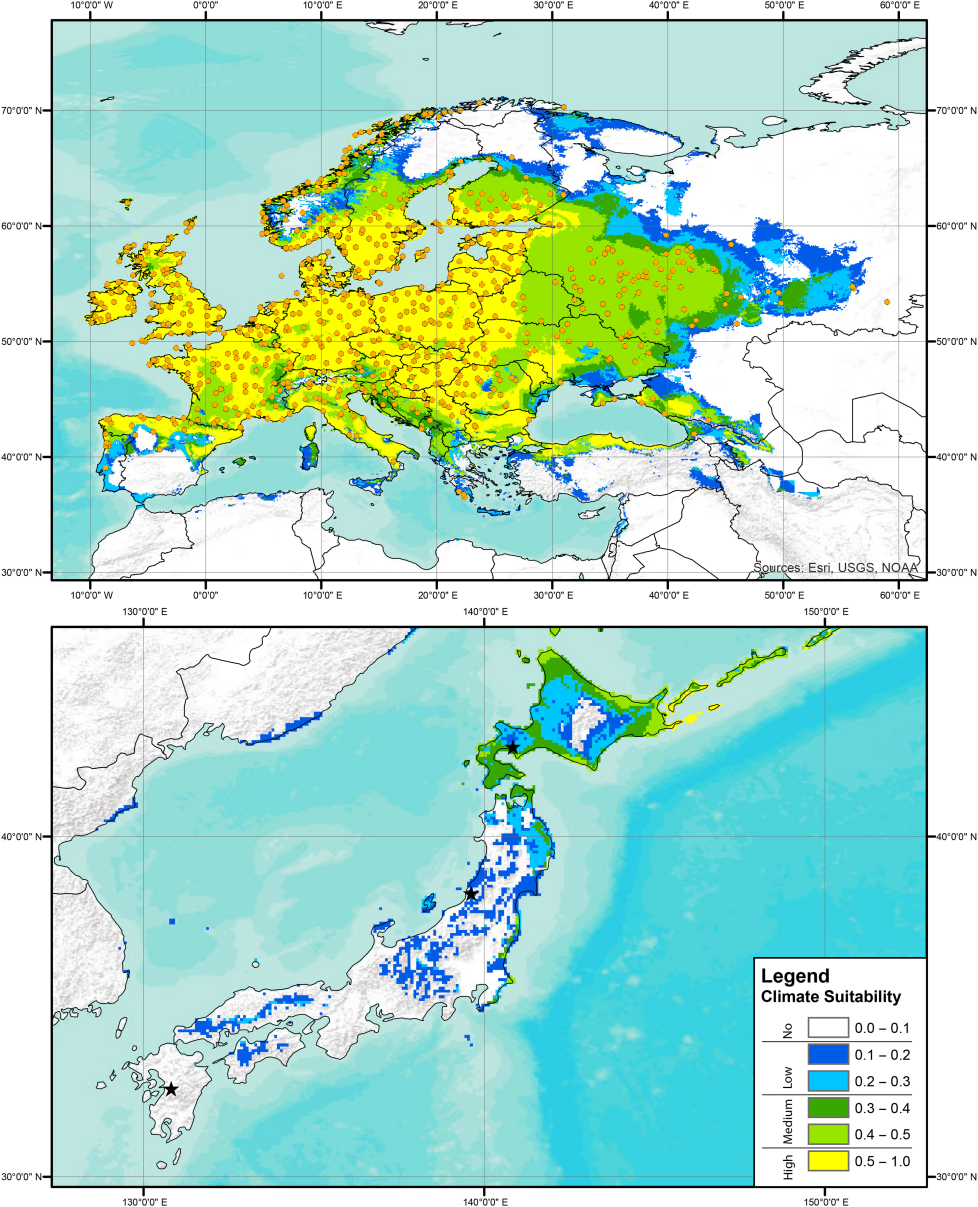
Climate suitability analysis results based on publicly available records of Japanese knotweed (*Reynoutria japonica*) from Europe in the GBIF database as estimated in MaxEnt. Orange circles represent the localities of samples used to construct the climate envelope. Stars represent the source localities of each of the strains of *Aphalara itadori* released or under review for knotweed biological control.

**FIGURE 2:**
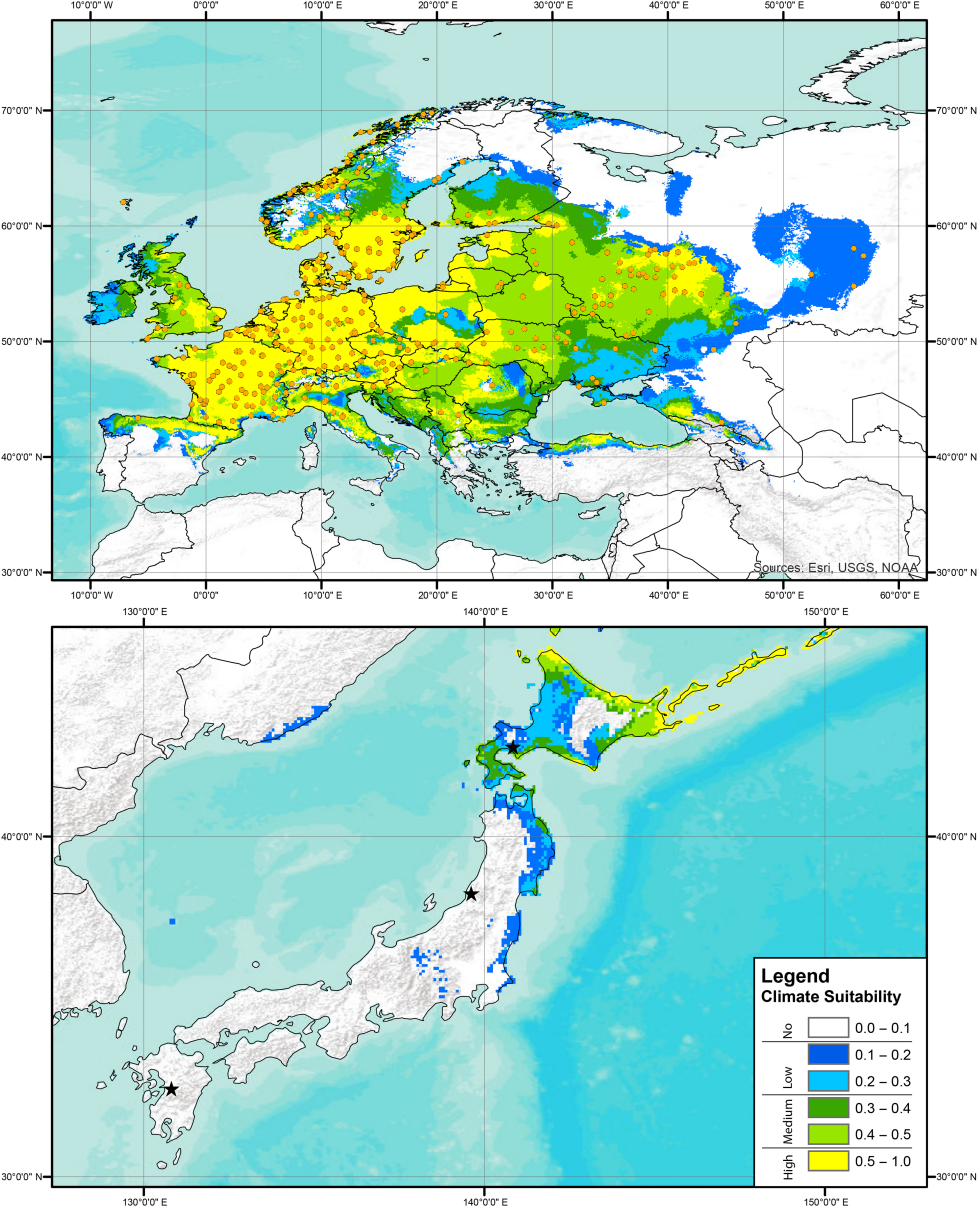
Climate suitability analysis as per Figure 1 based on publicly available records of Bohemian knotweed (*Reynoutria* × *bohemica*) from Europe. Stars represent the source localities of each of the strains of *Aphalara itadori* released or under review for knotweed biological control.

**FIGURE 3:**
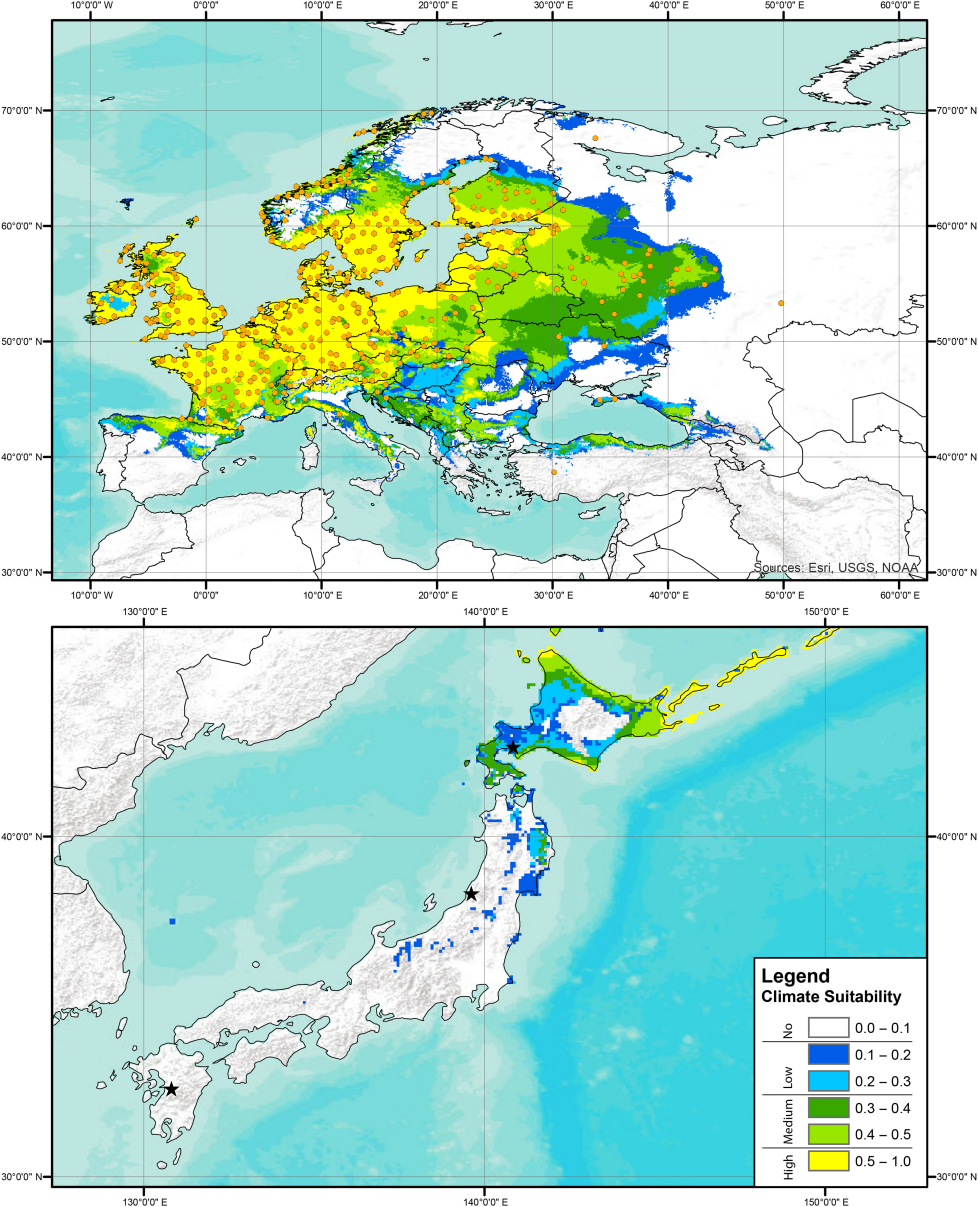
Climate suitability analysis as per Figure 1 based on publicly available records of Giant knotweed (*Reynoutria sachalinensis*) from Europe. Stars represent the source localities of each of the strains of *Aphalara itadori* released or under review for knotweed biological control.

**FIGURE 4:**
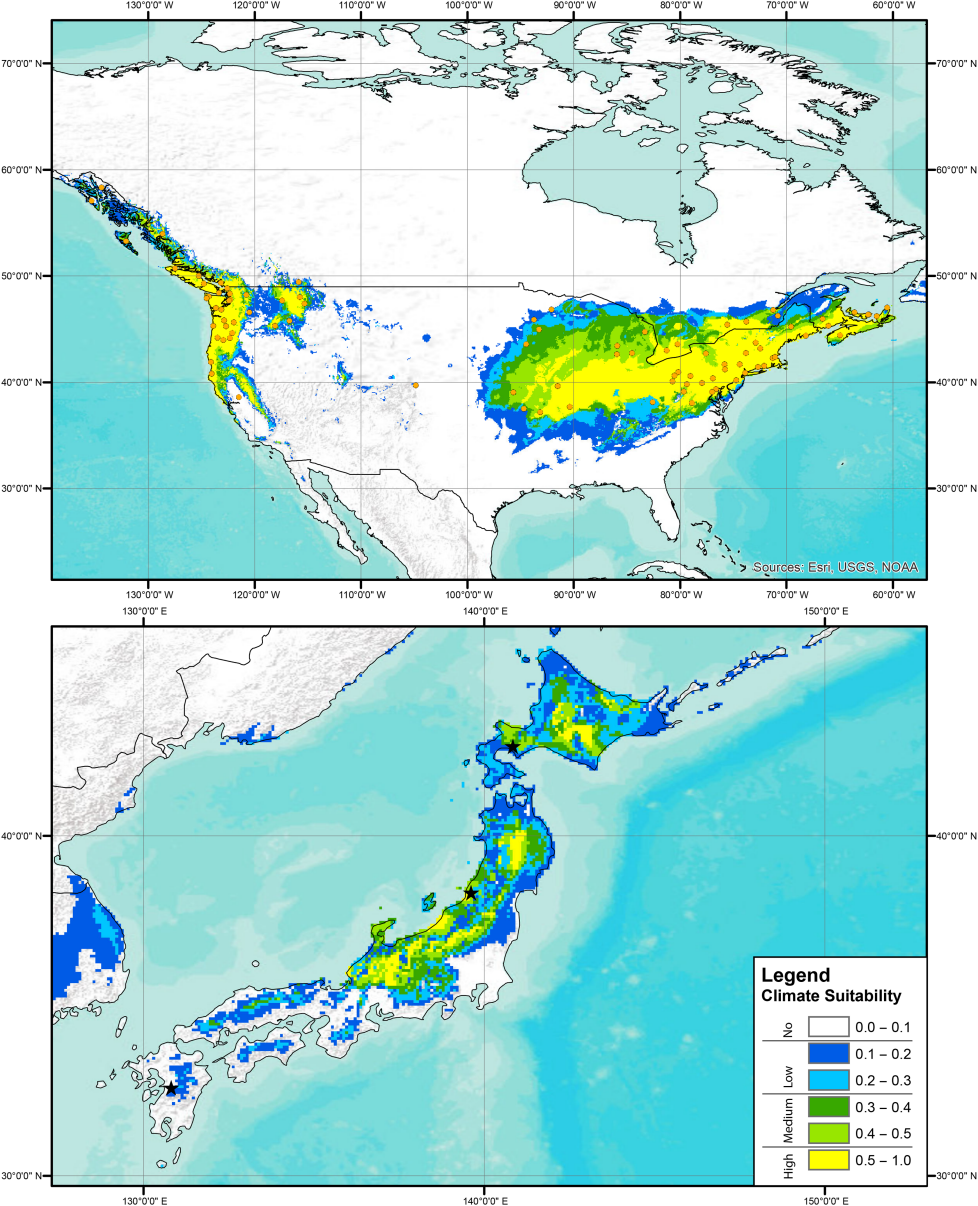
Climate suitability analysis as per Figure 1 based on publicly available records of Bohemian knotweed (*Reynoutria* × *bohemica*) from North America. Stars represent the source localities of each of the strains of *Aphalara itadori* released or under review for knotweed biological control.

**FIGURE 5:**
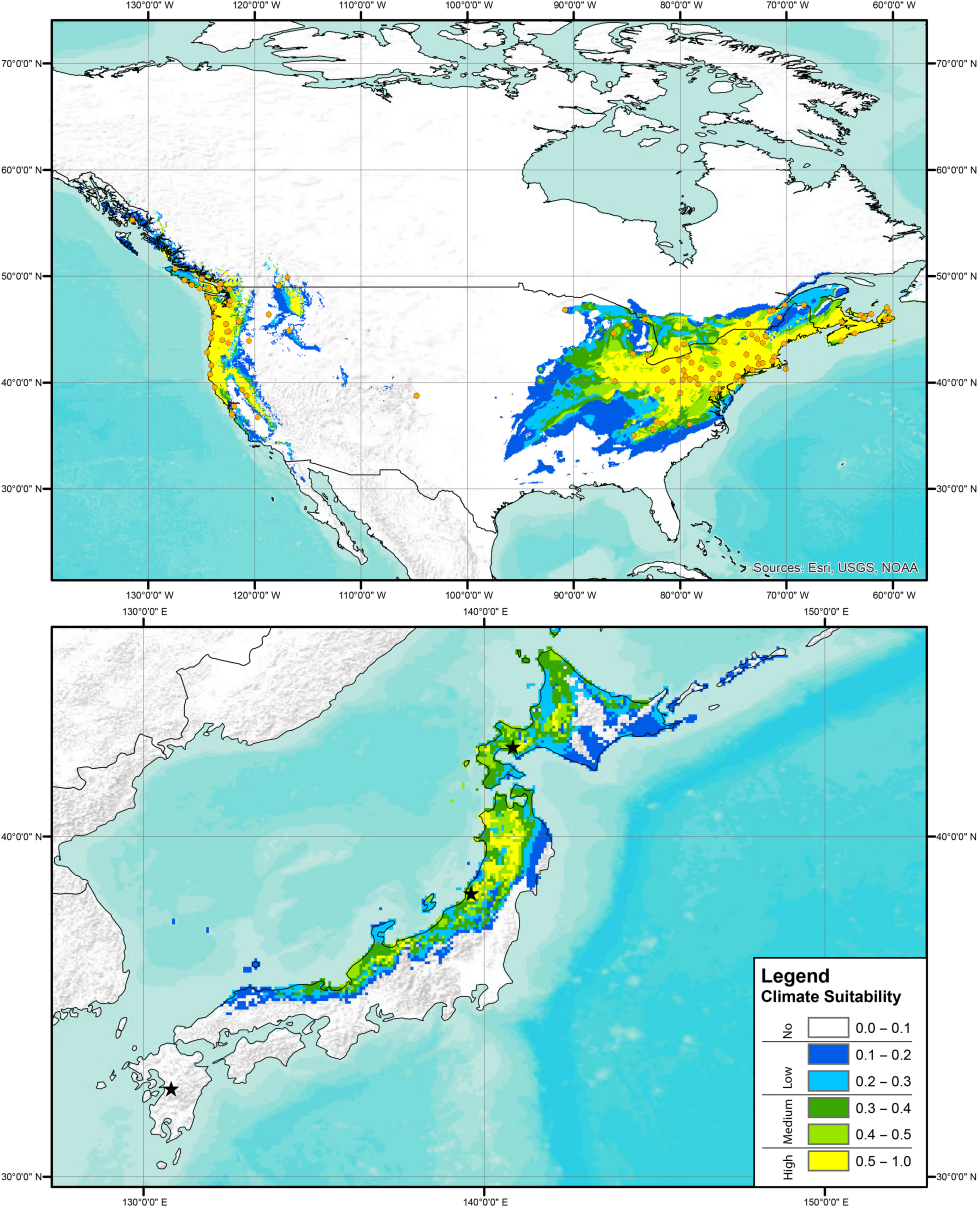
Climate suitability analysis as per Figure 1 based on publicly available records of Giant knotweed (*Reynoutria sachalinensis*) from North America. Stars represent the source localities of each of the strains of *Aphalara itadori* released or under review for knotweed biological control.

Predictions of climate suitability estimated from the European records indicated that the source locality of the Kyushu strain had no predicted suitability based on records for all three focal knotweed species, the source locality of the Murakami strain had no‐to‐low predicted suitability based on records of *R. japanoica* and no predicted suitability for based on records of *R*. × *bohemica* or *R. sachalinensis*, and the source locality of the Hokkaido strain had low‐to‐medium predicted suitability based on records for all three target knotweed species (Figures 1, 2, 3). A presentation of predicted climate suitability based on European records across a wider portion of central and east Asia are presented in Figures S1–S3. Predictions of climate suitability estimated from the North American records indicated that the source locality of the Kyushu strain had no‐to‐low predicted suitability based on records for *R*. × *bohemica* and no predicted suitability based on records for *R. sachalinensis*, the source locality of the Murakami strain had medium‐to‐high predicted suitability based on records for both *R*. × *bohemica* and *R. sachalinensis*, and the source locality of the Hokkaido strain had medium‐to‐high predicted suitability based on records for *R. achalinensis* and low‐to‐medium predicted suitability based on records for *R*. × *bohemica* (Figures 4 and 5). A presentation of predicted climate suitability based on North American records across a wider portion of central and east Asia are presented in Figures S4 and S5.

## 4 DISCUSSION

Understanding what factors promote and what factors inhibit the establishment of candidate biological control agents is critical if biological control is to transition from a reactionary science to a predictive one (Kimberling, 2004). There has, justifiably, been a major focus on predicting the potential for non‐target impacts (Barratt, 2011; Barratt et al., 2010), leading to extensive modeling of the potential extent of a candidate's introduced range based upon its native host's range (Barton, 2004; Kaser & Heimpel, 2015; Raghu et al., 2007). Increasingly, studies are conducting pre‐release ecological niche modeling to identify geographic regions from which candidate biological control agents might be selected (for some examples see Banerjee et al., 2019; Manrique et al., 2014; Mukherjee et al., 2011; Zalucki & van Klinken, 2006). These types of pre‐release comparisons can even help prioritize the suitability of different strains of biological control agents (Manrique et al., 2014), thus reducing both the environmental and political risks of introducing ineffective agents (McClay & Balciunas, 2005). Of course, these types of models are only estimates and make numerous assumptions to quantify and reduce complex biological and ecological processes. For example, models that integrate biological knowledge in addition to climatic variables produce more accurate models than those based on climate data alone (Low et al., 2020). In addition, the choice of climate variables can have important implications on results (see Booth, 2021). Furthermore, climate variables have been found to be more closely associated with some species compared to others, even when the species have overlapping distributions (Shabani et al., 2016). Most importantly, these approaches fail to account for the evolutionary potential of species (e.g., Bean et al., 2012; Diamond, 2018). Yet, despite these limitations, ecological niche models can prove useful as part of a larger discussion of factors that might influence species distributions (Warren, 2012). Here, we find that of the three strains of the biological control agent *Aphalara itadori* currently being considered or currently being released for the biological control of invasive knotweed species (*Reynoutria* spp.), only the Hokkaido strain is predicted to be suitable in both Europe and North America based on climate comparisons between the current distributions of knotweed species in these two regions and the source localities. The suitability of the other two strains differs by location and target species and is discussed in more detail below.

In our analyses and the analyses published in Andersen and Elkinton (2022), the Kyushu strain is found to have no‐to‐low‐climate suitability for any of the target knotweed species in either Europe or North America (Figures 4 and 5; Andersen & Elkinton, 2022). The lack of climate suitability of the Kysuhu strain mirrors field observations in North America, where to date, efforts to establish the Kyushu strain have been hindered by both biotic and abiotic factors (Andersen & Elkinton, 2022; Grevstad et al., 2022; Jones et al., 2020, 2021). In contrast, our analyses of the Murakami strain show that this agent has greater potential, particularly in North America. Analyses, based on the distributions of all three species of knotweed in North America, suggest that the Murakami strain has medium‐to‐high climate suitability in this region (Figures 4 and 5). In addition, laboratory testing has shown that this strain is capable of laying eggs on all three target species in a choice experiment and that feeding results in significant reductions for all three species in plant height (8% total reduction), and rhizome biomass for *R*. × *bohemica*, and *R. sachalinensis* (35% and 50%, respectively) (Camargo et al., 2022). Unfortunately, our climate analyses suggest that this strain has no‐to‐low suitability based on the distributions of all three target species in Europe (Figures 1, 2, 3). Analyses based on records of the Hokkaido strain suggest that this agent has at least some climate suitability based on knotweed records from both Europe and North America. We should note that this strain has been shown in the laboratory to have reduced fitness on species of knotweed other than *R. sachalinensis* (Grevstad et al., 2013); however, it is possible that additional populations feeding on the other target knotweed species might be present on Hokkaido as well, and we encourage further investigations in this region.

In an effort to create datasets with large enough geographic distributions to be statistically meaningful, these continental‐wide analyses might mask more localized locations where climate suitability of the different strains might be achieved. For example, despite our previous findings that the Kyushu strain has no‐to‐low‐climate suitability for most of North America against *R. japonica* (Andersen & Elkinton, 2022), in the spring of 2022 we did note the presence of 15 overwintering adults at one field release site in western Massachusetts (Andersen & Elkinton, unpublished data). Similar reports of small numbers of overwintering adults of the Kyushu strain were also reported in coastal Rhode Island (Dr. Lisa Tewksbury, personal communication) and in North Carolina (Dr. Fritzi Grevstad, personal communication), and individuals of the Murakami strain have been observed persisting and dispersing in the field in the Netherlands (Dr. Suzanne Lommen, personal communication), despite our results suggesting they have no or low‐climate suitability. While records from several more years will be necessary before we can consider these localized populations “established,” they do suggest that even in areas where suitability might be predicted to be low based on our analyses, that persistence and eventually establishment might be possible.

Lastly, we would like to point out an interesting result from our analyses. Our climate models, based on the invasive distributions of each knotweed species, tend to predict low‐climate suitability to areas across much of the Japanese archipelago. On one hand, readers should interpret this result as an indication that our ecological niche models are capturing only a portion of the factors that shape the potential and realized niches of an organism (as reviewed above). On the other hand, we believe this result highlights the fact that local adaptation has occurred in this system. Invasive knotweeds have been present in North America and Europe for nearly 200 years (Conolly, 1977), and that during that time they have successfully adapted to the local environments in their introduced ranges—this evolutionary potential is likely one of the reasons that they are listed among worlds 100 most invasive species (IUCN, 2021). Given that coevolutionary forces form the basis of sustainable biological control services (Holt & Hochberg, 1997), this potential mismatch between the newly evolved realized niche of an invasive species and the existing potential niche of the natural enemy in its native range, such as *A. itadori*, could have profound implications on the “success” of a biological control program if the target and the natural enemy no longer share the same climatic constraints.

## AUTHOR CONTRIBUTIONS

**Jeremy C. Andersen:** Conceptualization (equal); data curation (equal); formal analysis (lead); funding acquisition (equal); investigation (equal); methodology (equal); project administration (equal); resources (equal); software (equal); supervision (equal); validation (equal); visualization (equal); writing – original draft (equal); writing – review and editing (equal). **Joseph S. Elkinton:** Conceptualization (equal); data curation (equal); formal analysis (equal); funding acquisition (equal); investigation (equal); methodology (equal); project administration (equal); resources (equal); software (equal); supervision (equal); validation (equal); visualization (equal); writing – original draft (equal); writing – review and editing (equal).

## FUNDING INFORMATION

This research was funded by a United States Department of Agriculture Animal and Plant Health Inspection Service (USDA‐APHIS) co‐operative agreement (USDA‐APHIS‐10025‐PPQFO000‐20‐0091) awarded to J.S.E.

## CONFLICT OF INTEREST STATEMENT

The authors declare no conflicts of interest.

## Supporting information


Figures S1–S5

## Data Availability

GBIF occurrence data are available at https://doi.org/10.15468/dl.pdjdh8
