# Supplementary material for: Climate suitability analyses compare the distributions of invasive knotweeds in Europe and North America with the source localities of their introduced biological control agents
Source: Ecol Evol. 2023 Sep 12;13(9):e10494. doi: 10.1002/ece3.10494 (PMC10495814; doi:10.1002/ece3.10494)
Supplement: Supplementary file 1 — Figures S1–S5 [file ECE3-13-e10494-s001.pdf]

Supplemental Figures: Andersen and Elkinton 2023, Climate suitability analyses compare the distributions of invasive knotweeds in Europe and North America with the source localities of their introduced biological control agents.

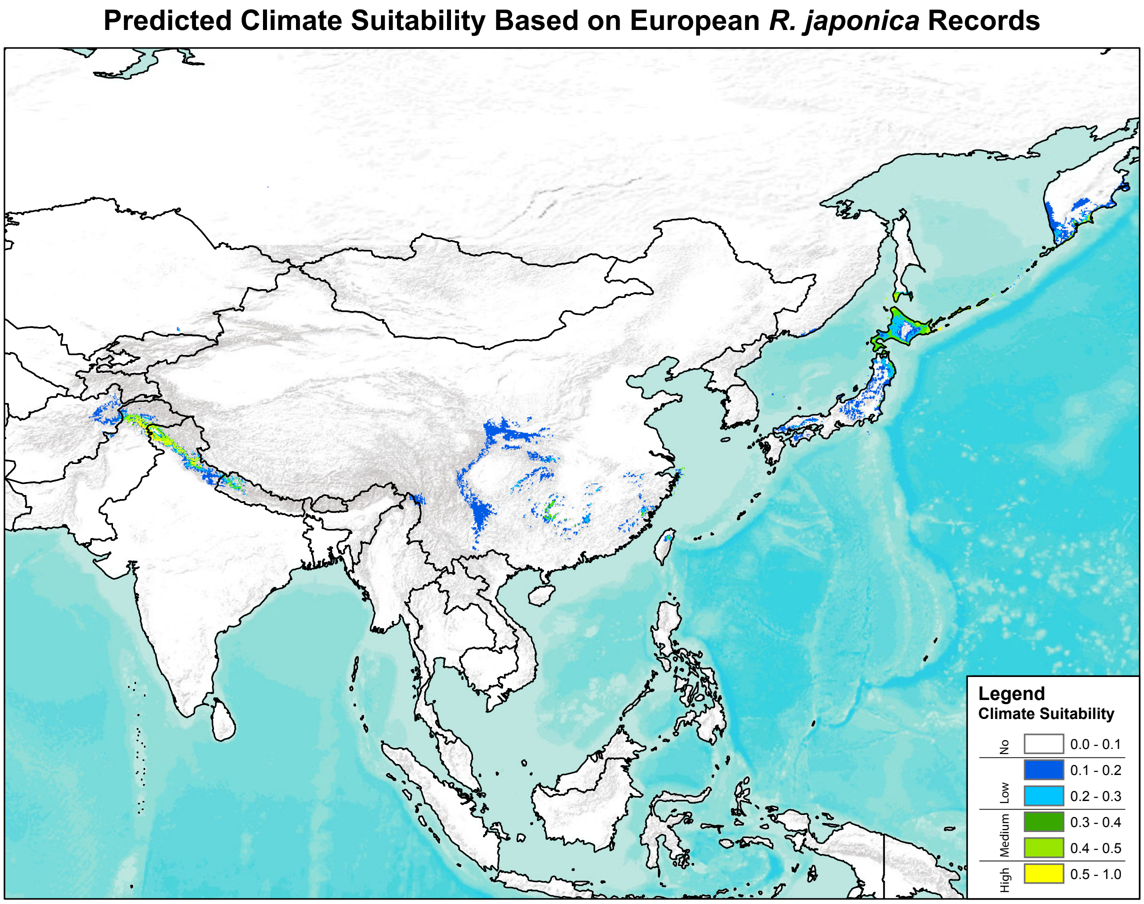

Supplemental Figure S1. Climate suitability analysis results based on publicly available records of Japanese knotweed (*R. japonica*) from Europe in the GBIF database as estimated in MaxEnt. Orange circles represent the localities of samples used to construct the climate envelope.

Supplemental Figures: Andersen and Elkinton 2023, Climate suitability analyses compare the distributions of invasive knotweeds in Europe and North America with the source localities of their introduced biological control agents.

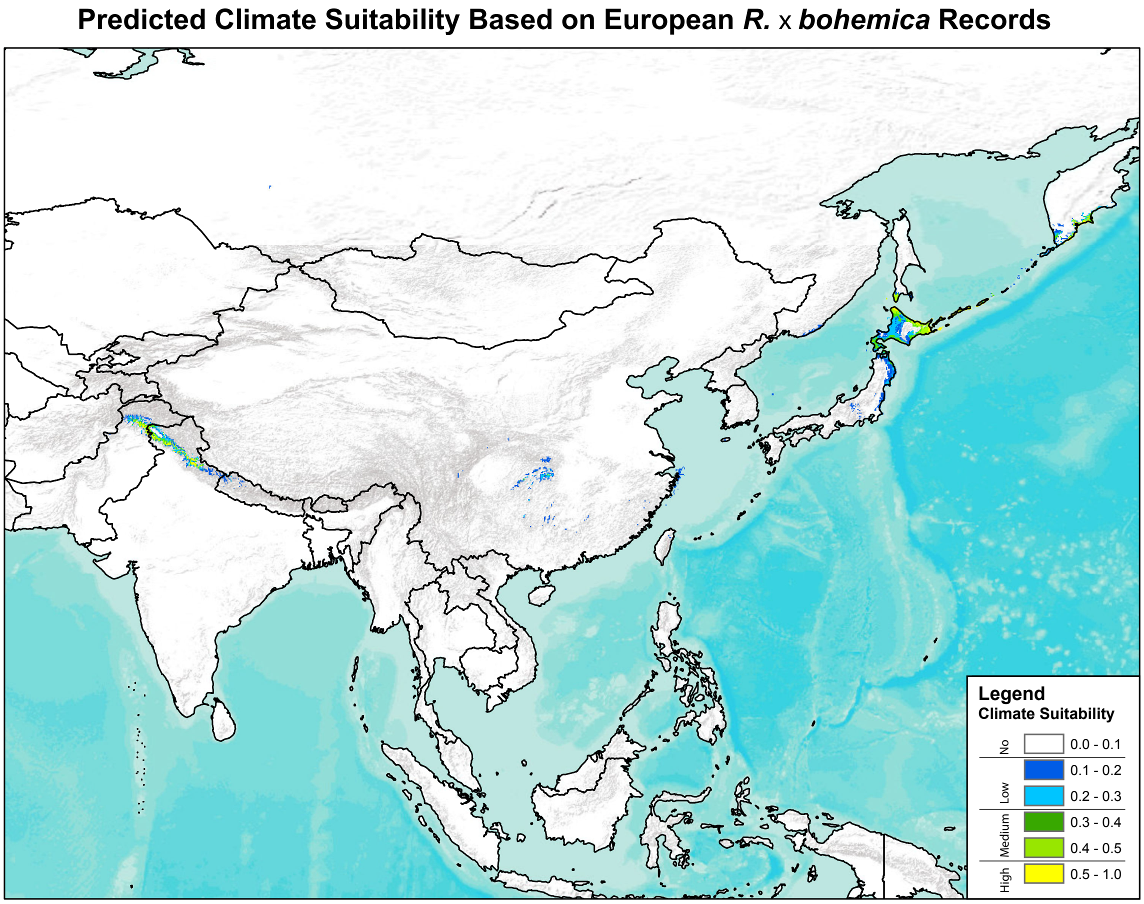

Supplemental Figure S2. Climate suitability analysis as per Supplemental Figure S1 based on publicly available records of Bohemian knotweed (*R.* × *bohemica*) from Europe.

Supplemental Figures: Andersen and Elkinton 2023, Climate suitability analyses compare the distributions of invasive knotweeds in Europe and North America with the source localities of their introduced biological control agents.

Predicted Climate Suitability Based on European *R. sachalinensis* Records

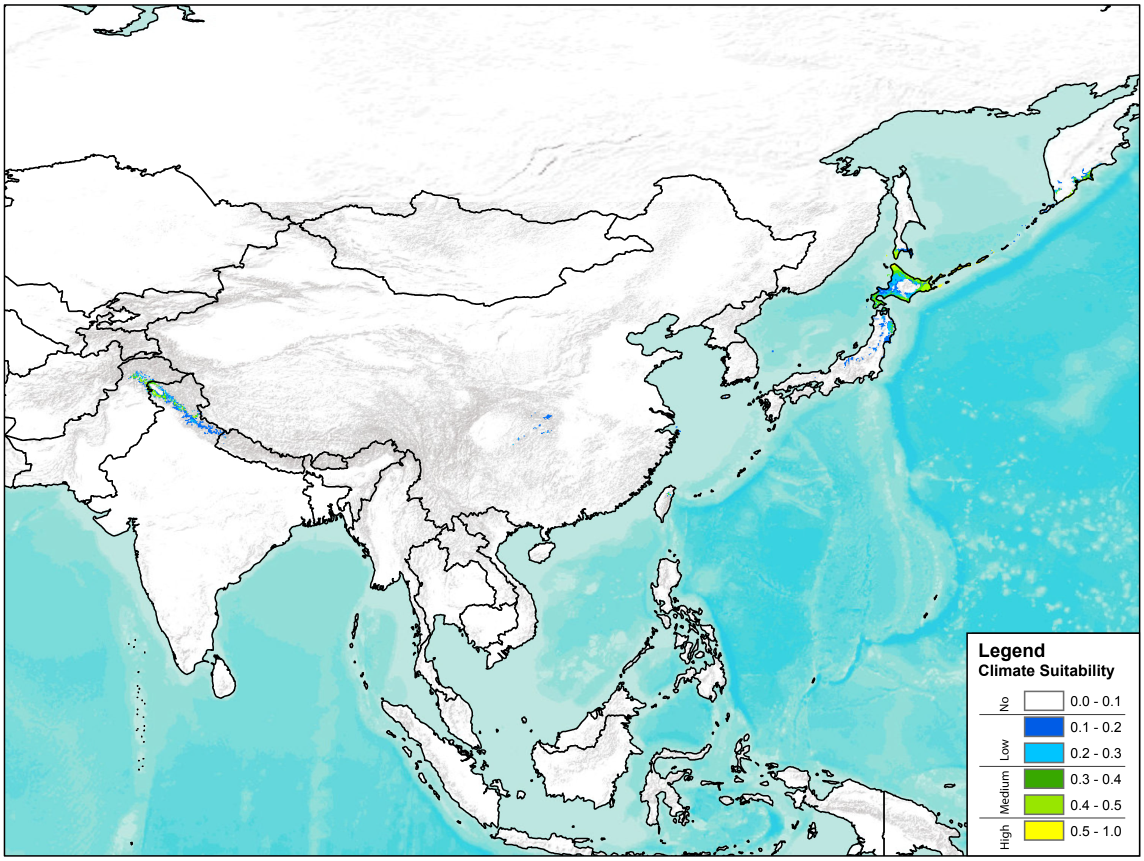

Supplemental Figure S3. Climate suitability analysis as per Supplemental Figure S1 based on publicly available records of Giant knotweed (*R. sachalinensis*) from Europe.

Supplemental Figures: Andersen and Elkinton 2023, Climate suitability analyses compare the distributions of invasive knotweeds in Europe and North America with the source localities of their introduced biological control agents.

Predicted Climate Suitability Based on North American *R.* x *bohemica* Records

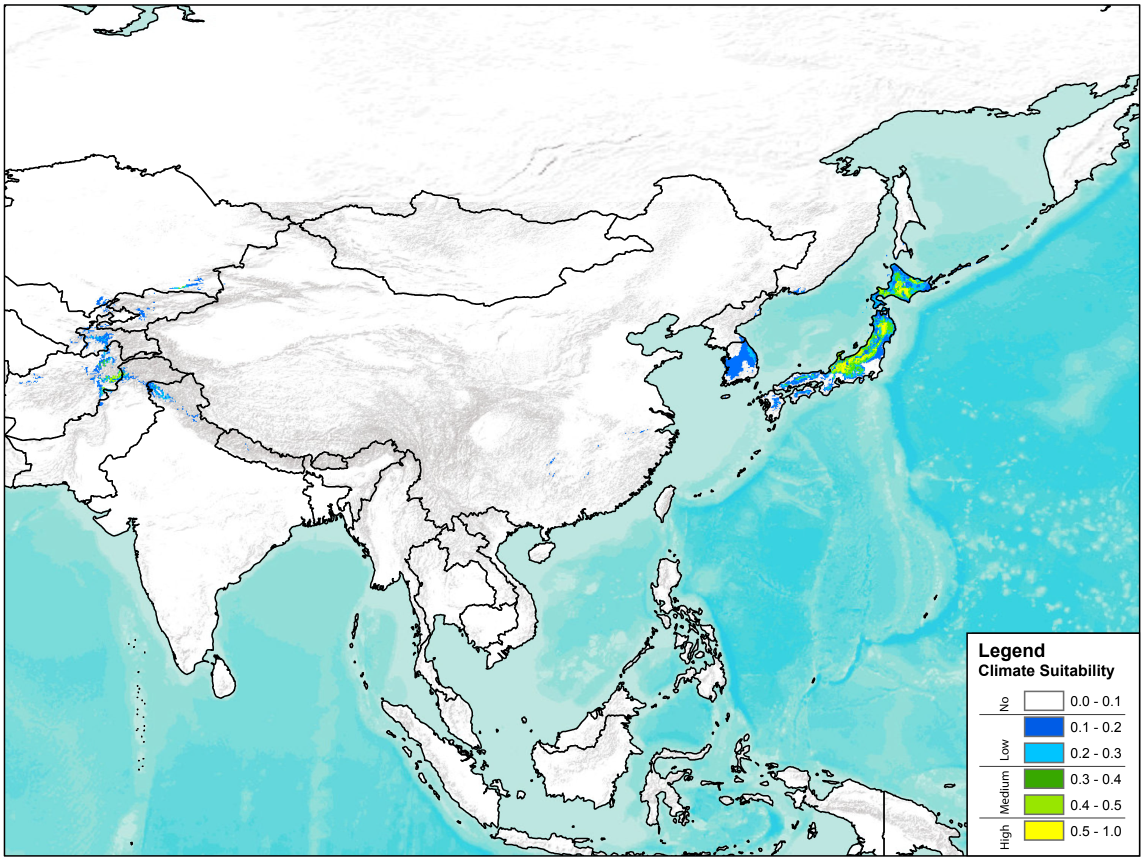

Supplemental Figure S4. Climate suitability analysis as per Supplemental Figure S1 based on publicly available records of Bohemian knotweed (*R.* × *bohemica*) from North America.

Supplemental Figures: Andersen and Elkinton 2023, Climate suitability analyses compare the distributions of invasive knotweeds in Europe and North America with the source localities of their introduced biological control agents.

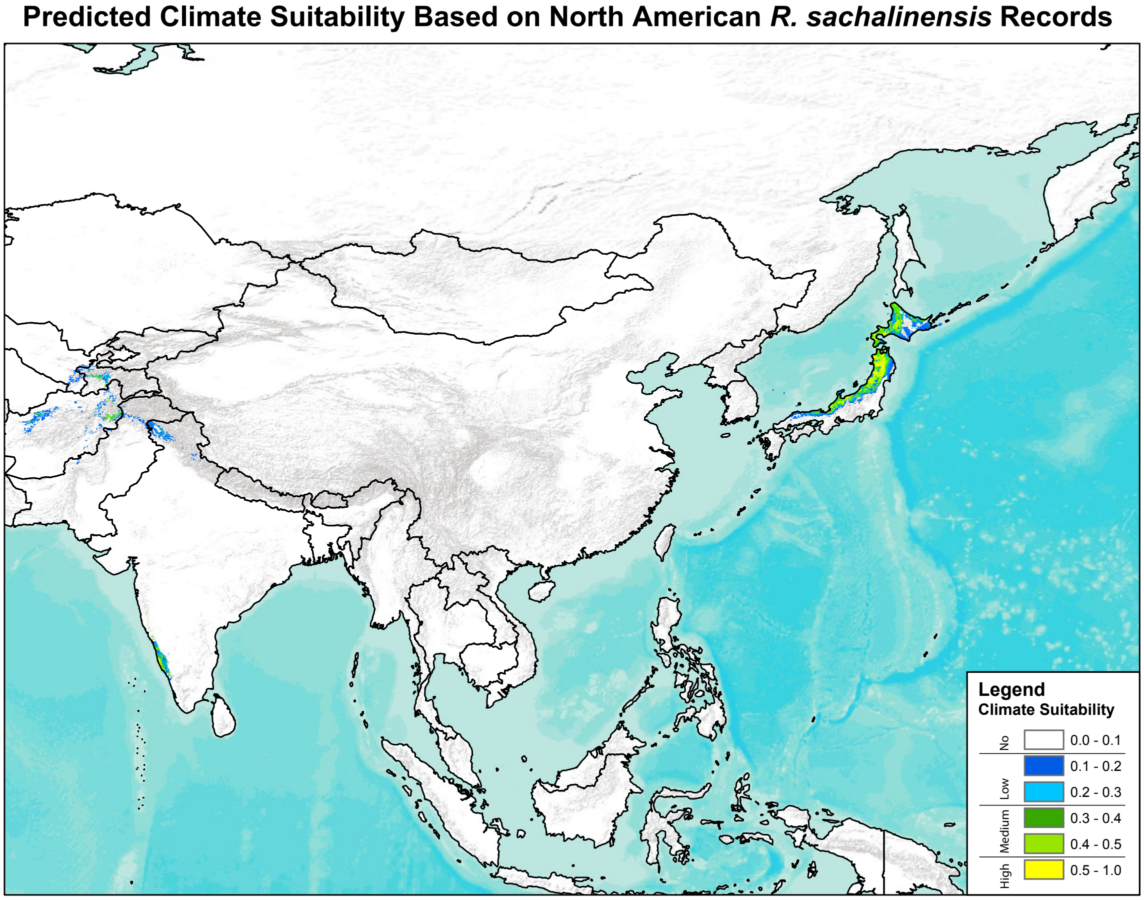

Supplemental Figure S5. Climate suitability analysis as per Supplemental Figure S1 based on publicly available records of Giant knotweed (*R. sachalinensis*) from North America.
